# Establishment and Clinical Application of a RPA-LFS Assay for Detection of Capsulated and Non-Capsulated *Haemophilus influenzae*

**DOI:** 10.3389/fcimb.2022.878813

**Published:** 2022-04-21

**Authors:** Yan Wang, Aibo Liu, Mei Fu, Jingjing Guo, Lei Wang, Xiaohua Zuo, Fenfen Ma

**Affiliations:** ^1^ Department of Medicine Laboratory, Department of Cardiac Function Examination, The Second People's Hospital of Lianyungang (Cancer Hospital of Lianyungang), Lianyungang, China; ^2^ Department of Laboratory Medicine, Sichuan Provincial People’s Hospital, University of Electronic Science and Technology of China, Chengdu, China; ^3^ Department of Medicine Laboratory, Xuzhou Central Hospital, Xuzhou, China; ^4^ Department of Pain Management, The Affiliated Huai’an Hospital of Xuzhou Medical University and The Second People’s Hospital of Huai’an, Huai’an, China; ^5^ Department of Cardiac Function Examination, The Second People’s Hospital of Lianyungang, Lianyungang City, China

**Keywords:** *H. influenzae*, RPA-LFS, capsular, non-encapsulated, dual PCR

## Abstract

A recombinase polymerase amplification-lateral flow strip assay was established for detection of the outer membrane protein P6 (*omp6*) and the capsule encoding gene *bexA* of *Haemophilus influenzae* and the detection limit, sensitivity, and specificity were determined. Specific primers and probes were designed based on the published nucleotide sequences of *omp6* and *bexA*. The minimum detection limit was determined with standard strains and the practical applicability of the RPA-LFS assay was assessed by detection of 209 clinical samples. The results confirmed that the RPA-LFS assay was both specific and sensitive for the detection of capsulated and non-capsulated *H. influenzae* with a detection limit of 1 CFU/µL. The detection rate of the 209 clinical samples was 97.1%, while the detection rate of capsulated *H. influenzae* was 63.2%. The detection results were consistent with the traditional culture method and dual polymerase chain reaction (PCR), confirming the applicability of the RPA-LFS assay.

## Introduction

*H. influenzae* is a Gram-negative bacteria that is responsible for about 3 million cases of pneumonia, meningitis, and otitis media annually worldwide, with most infections occurring in children aged ≤ 5 years (Ali et al., 2018; Biondi et al., 2019; Silva et al., 2021; Giufrè et al., 2022). *H. influenzae* is divided into capsulated and non-capsulated strains, while capsulated *H. influenzae* is further divided into six serotypes (a–f) (Wen et al., 2020; Fuji et al., 2021). Although the distribution of serotypes varies greatly among different regions, *H. influenzae* type b is the most pathogenic and can cause severe meningitis and sepsis (Moxon and Kroll, 1988; Gessner, 2002; Guellil et al., 2022; Nolen et al., 2022). Routine immunization with Hib conjugate vaccines has significantly reduced the incidence of Hib-related diseases in developed countries, although the incidence remains relatively high in underdeveloped regions (Adegbola et al., 2014; Langereis and de Jonge, 2015). At present, traditional bacterial culture is the gold standard for the detection of *H. influenzae* (Farajzadeh Sheikh et al., 2021). However, *H. influenzae* is a fastidious bacterium that requires special growth factors, long-term culture, and complicated operation procedures, thus the isolation rate remains relatively low, which delays diagnosis and treatment. Hence, a rapid and accurate method for detection of *H. influenzae* is urgently needed.

With the rapid development of molecular diagnostic technologies, PCR is widely used for the detection of various microorganisms (Takahashi et al., 2021; Boukharouba et al., 2022; Serigstad et al., 2022). A PCR method was developed for the detection of *H. influenzae* based on the 16S rRNA gene. Although the sensitivity for detection of *H*. *influenzae* is reportedly 97.53%, this method cannot distinguish capsulated from non-capsulated *H. influenzae* (Tian et al., 2008). Another PCR method was established for the detection of capsulated and non-capsulated *H. influenzae* based on the *omp6* and *bexA* genes, but requires expensive equipment and trained technicians (Tian et al., 2008; Abdeldaim et al., 2010). In order to reduce the dependence on instruments and professional technicians, an alternative method for the detection of *H. influenzae* was developed using multiple cross displacement amplification and a nanoparticle-based lateral flow biosensor based on the *omp6* gene with a reaction for 1 h at 58–65°C (Cao et al., 2021). Although this method does not require expensive equipment, primer design is complicated, the reaction time is relatively long, and false-positive results are problematic.

RPA is an emerging isothermal amplification technology with improved specificity, sensitivity, and portability than other methods (Wang et al., 2019; Wang et al., 2021b). RPA uses recombinase to open the DNA double strand allowing the primer to bind to the target fragment and the polymerase Bsu with strand displacement activity to recognize the 3’ end of the primer for stable amplification (Piepenburg et al., 2006; Daher et al., 2016). The reaction is conducted for 20 min at 30–45°C and does not require sophisticated instruments or professional technicians. The amplification products can be obtained by gel electrophoresis, fluorescence detection, and colloidal gold test strips. Different from gel electrophoresis and fluorescence detection methods, colloidal gold test strips use the principle of antigen-antibody binding to detect amplification products (Wu et al., 2020; Yang et al., 2020). By adding a probe labeled with fluorescein isothiocyanate (FITC) at the 5’ end to the RPA reaction system and labeling the 5’ end of the reverse primer with biotin, the amplification products can be combined with a specific antibody at the detection line on the colloidal gold test strip as a colorimetric indicator. The combination of RPA and colloidal gold test strips further improved the reaction time and specificity of this technology. It also increases the expectation that it can meet the rapid and timely on-site testing in medically underserved areas. Therefore, RPA-LFS assays have been developed for the detection of various pathogens, including *Pseudomonas aeruginosa*, *Candida albicans*, and *Listeria monocytogene*s (Wang et al., 2019; Yang et al., 2021a; Wang et al., 2021a).

In this study, an RPA-LFS assay was established for the identification of capsulated and non-capsulated *H. influenzae* based on the *omp6* and *bexA* genes, as a rapid, sensitive, and portable detection system.

## Materials and Methods

### Ethics Statement

The study protocol was approved by the Medical Ethics Committee of the Second People’s Hospital of Lianyungang City (Lianyungang, China; approval no: 2020005). The clinical strains were isolated from sputum and nasal swab samples collected from 2020 to 2022. All patients agreed the use of the samples in this study and completed a written consent form.

### Preparation of Bacterial Strains and Clinical Samples

Two strains of *H. influenzae* (non-capsulated, American Type Culture Collection ATCC 49247; and capsulated, ATCC 9334) were purchased from Shanghai Covey Chemical Technology Co., Ltd. (Shanghai, China). The specificity of the RPA-LFS assay was investigated based on the detection of the *omp6* gene of 20 *H. influenzae* isolates from sputum (ten non-capsulated and ten capsulated) along with 23 other common pathogenic bacteria provided by our laboratory (i.e., *Acinetobacter calcoaceticus*, *Acinetobacter lwoffi*, *Acinetobacter haemolytius*, *Acinetobacter junii*, *Acinetobacter johnsonii*, *Candida albicans*, *Enterobacter cloacae*, *Enterococcus faecium*, *Escherichia coli* O157, *Mycobacterium tuberculosis* H37Ra, *Pseudomonas aeruginosa*, *Staphylococcus aureus*, *Staphylococcus capitis*, *Staphylococcus epidermidis*, *Staphylococcus haemolyticus*, *Staphylococcus hominis*, *Staphylococcus saprophyticus*, *Staphylococcus warneri*, *Stenotrophomonas maltophilia*, *Streptococcus pneumonia*, *Viridans streptococci*, *Klebsiella pneumoniae*, and *Acinetobacter baumannii*; **Table 1**). In total, 209 samples (sputum or nasal swab) were collected from patients with suspected *H. influenzae* infection in four hospitals located in Lianyungang and other cities (i.e., Lianyungang Second People’s Hospital, Sichuan Provincial People’s Hospital, Xuzhou Central Hospital, The Second People’s Hospital of Huai’an). All bacterial samples were incubated for 10 min at 100°C and, if not otherwise specified, 1 µL of the heat-treated culture at 10^5^ CFU/mL was used as the template.

**Table 1:** Primers and probes.

Primers/Probes	Primer Sequences	Size (bp)	Reaction name
Omp6-F1	ACACTGATGAACGTGGTACACCAGAATACAA	31	RPA
Omp6-R1	ACCAGCTAAATAACCTTTAACTGCATCTGCA	31	
Omp6-F2	CAAACTTTTGGCGGTTACTCTGTTGCTGATC	31	
Omp6-R2	TGCGTCTAAGATTTGAACGTATTCACCAGTA	31	
bexA-F1	CGGTTGAGTTTGATTGTTATTTAATTGATGAG	32	
bexA-R1	TGTGAAACTAAAATGATAGAACGGTCTTTGC	31	
bexA-F2	TCTATCATTTTAGTTTCACATAGCCCGAGTG	31	
bexA-R2	TGTAGTATTGATACGCTTTGTCCATGTCTTC	31	
Omp6-P	FITC-ACACTGATGAACGTGATACACCATAATACAA[THF]ATCGTATTAGGCCAA-C3 spacer	46	RPA-LFS
Omp6-R1B	Biotin-ACCAGCTGAGTAACCTTTAACTAGATCTGCA	31	
Omp6-F3	CAGGAAATGGTGCTGCTCAAACTTTTGGCGGTTAC	35	
bexA-P	FITC-CGGTTGAGTATGATTGTTATGTAATTGATGAG[THF]TGATTGTAGTAGGG-C3 spacer	46	
bexA-R1B	Biotin-TGTGAAACGAAAATGATAGAACGGTCTTTGC	31	
bexA-F3	CAGGAAATGGTGCTGCTCAAACTTTTGGCGGTTAC	35	
Omp6-F	ATGAACAAATTTGTTAAATCA	21	Dual PCR
Omp6-R	TGCGATGTTGTATTCAGGTGTA	22	(Zhao et al., 2017)
bexA-F	CGTTTGTATGATGTTGATCCA	21	
bexA-R	TGTCCATGTCTTCAAAATG	19	

F, forward primer; R, reverse primer; P, probe.

### Primers and Probes

Two RPA primers were designed based on the sequences of the *omp6 and bexA* genes with Primer Premier 5.0 software (Premier Biosoft, Palo Alto, CA, USA). The minimum and maximum product sizes of the primers were set at 100 and 300 bp, while the minimum and maximum primer sizes were 30 and 35 bp, respectively. Primers with sequence pairing of more than three consecutive bases (and more than one base at the 3ʹ end) were abandoned. The sequences of the primers and probes were confirmed for species specificity using the Primer-Basic Local Alignment Search Tool (https://www.ncbi.nlm.nih.gov/tools/primer-blast/).

### RPA Procedure

RPA reactions were performed using the TwistAmp^®^ Liquid DNA Amplification Kit (TwistDx Inc., Maidenhead, UK) in accordance with the manufacturer’s instructions. Each 50-µL reaction contained 25 μL of 2× reaction buffer, 5 μL of 10× Basic e-mix, 2.5 μL of 20× core mix, 2.4 μL of 10 μM forward primer, 2.4 μL of 10 μM reverse primer, and 9.2 μL of distilled water. In addition, 2.5 µL of 280 mM magnesium acetate and 1 μL of the template were added to the lid of the reaction tube. After brief centrifugation, the reaction mixture was incubated for 30 min at 37°C. The RPA amplification products were purified using a PCR Cleaning Kit (Shanghai Meiji Biotechnology Co., Ltd., Shanghai, China) and separated by electrophoresis on a 2% agarose gel.

### Probe Design

Primer Premier 5 software was used to design specific probes between the forward and reverse primer targeting sequences of the *omp6* and *bexA* genes. The formation of dimers, hairpin structures, and mismatches between the probe and reverse primer were theoretically avoided as much as possible by adhering to the following parameters: probe size, 46–51 bp; GC content, 20%–80%; melting temperature, 57–80°C; maximum hairpin score, 9; maximum primer-dimer score, 9; maximum poly-X, 5; and other parameters, default values. In addition, the 5’ end of the probe was labeled with FITC, the 3’ end was blocked with SpC3, the middle base of the probe was replaced with tetrahydrofuran (THF) with at least 30 bp before the THF site and 15 bp after, and the 5’ end of the reverse primer was labeled with biotin.

### Procedure for the RPA-LFS Assay

The RPA reactions were conducted using the TwistAmp^®^ DNA Amplification nfo Kit (TwistDx). Each reaction mixture consisted of 29.5 μL of rehydration buffer, 2.1 μL of 10 μM forward primer, 2.1 μL of 10 μM reverse primer, 0.6 μL of 10 μM probe, and 12.2 μL of distilled water. To initiate the reaction, 1 μL of the template and 2.5 μL of 280 mM magnesium acetate were added to the mixture. After brief centrifugation, the reaction mixture was incubated for 5–35 min at 30–45°C.

Owing to the remarkable sensitivity of colloidal gold test strips, only a small amount of product is needed for detection, thus appropriate dilutions might be required. Only 2 µL of the amplification products were used for LFS detection (Ustar Biotechnologies Ltd., Hangzhou, China). The amplification products were added to the sample pad of the LFS, while the stick of the LFS was inserted into 100 μL of the sample buffer (Ustar Biotech) for 2 min prior to visualizing the results.

### Detection Limit of the RPA–LFS Assay

Standard capsulated (ATCC 9334, type b) and non-capsulated *H. influenzae* were inactivated by 10-fold serial dilution with ddH_2_O (10^6^–10^0^) for use as templates for the RPA-LFS assay. To determine whether contamination with other strains would interfere with detection sensitivity, 10^5^ CFU/μL of heat-treated *S. pneumoniae* were added to 10-fold dilutions of heat-treated capsulated and non-capsulated *H. influenzae* culture (10^5^–10^0^ CFU/μL), respectively.

### Dual PCR Assay

Primers for the dual PCR assay based on the *omp6* and *bexA* genes were used as the control group in this experiment (**Table 1**). Each 50-μL reaction contained 2.5 M deoxynucleoside triphosphates, 250 nM upstream and downstream primers for the *omp6* and *bexA* genes, 2.5 U of Ex-Taq DNA polymerase (TaKaRa Biotechnology Co., Ltd., Dalian, China), 2 μL of the template, and 1 × PCR buffer (pH = 8.3). The PCR cycling conditions consisted of an initial denaturation step at 94°C for 4 min, followed by 35 cycles at 94°C for 30 s, 54°C for 30 s, and 72°C for 45 s, and a final extension step at 72°C for 5 min. The amplification products were separated by electrophoresis on a 2% agarose gel that was prestained with 1 μg/mL of ethidium bromide.

### Evaluation of the RPA-LFS Assay With Clinical Specimens

The practical application of the RPA-LFS assay was verified by comparisons to the results of the dual PCR assay established by Zhao et al. with 209 clinical specimens.

## Results

### Design and Screening of Primers for *H. influenzae* Detection

**Figure 1:**
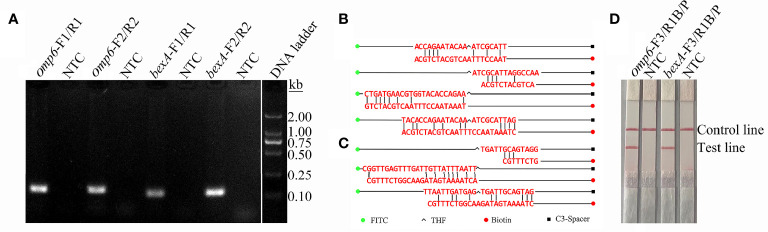
Screening of primers and probes. **(A)** RPA was performed to screen the primers. Agarose gel images showing the amplification results of primer pairs targeting the two virulence genes *omp6* and *bexA*. The primer pair is indicated at the top of each lane. NTC, no template control. The band sizes of the DNA ladder are shown on the right. The images represent the results of three independent experiments. **(B)** A dimer formed between probe-reverse primer and probe-probe based on the *omp6* gene design. **(C)** Dimers formed between the probe-reverse primer and probe-probe based on the *bexA* gene design. **(D)** Testing of the modified primer–probe set. The image shows the LFS results of RPA amplifications. The name of each primer-probe set is indicated at the top of the corresponding strip. The template consisted of 1 µL of boiled non-capsulated or capsulated *H. influenzae* culture at 10^6^ CFU/mL. NTC, no template control. The positions of the test and control lines are marked on the right of the strip image. The reactions were performed for 30 min at 37°C. The image represents the results of three independent experiments.

In order to distinguish the capsulated from non-capsulated *H. influenzae*, two pairs of primers were designed based on the conserved regions of the *omp6* and *bexA* genes (**Table 1**). As shown in **Figure 1A**, each primer pair amplified the corresponding target strain, and except for the target band, there were no non-specific bands or primer dimers. However, as indicated by the gel map, the bands amplified with the primer pairs *omp6*-F1/R1 and *bexA*-F2/R2 were notably brighter, indicating greater amplification efficiency. Therefore, the primer pairs *omp6*-F1/R1 and *bexA*-F2/R2 were selected for further experimentation.

### Addition of Probes to the RPA-LFS Reaction

The introduction of probes into the RPA system not only improves the specificity and sensitivity of the reaction, but also reduces the generation of primer-dimers. In order to increase specificity and sensitivity, corresponding probes were designed based on the target regions of the primer pairs *omp6*-F1/R1 and *bexA*-F2/R2. Although the introduction of probes can reduce the generation of primer dimers, false-positive signals are unavoidable due to the formation of dimers between the probe and reverse primer with stable amplification ability. The formation of dimers formed between the probe and reverse primer was detected using Primer Premier 5 software. As shown in **Figure 1B**, each probe and the corresponding reverse primer have a certain complementary fragment and some cover the THF site or expose the reverse 3’-OH end of the reverse primer. These dimers can be stably amplified, resulting in false-positive signals. RPA can tolerate a certain extent of base mismatch with no effect on the amplification efficiency (Daher et al., 2016; Liu et al., 2019). Nonetheless, many previous studies successfully established RPA-LFS assays (Yang et al., 2020; Yang et al., 2021b). In order to avoid false-positive results, mismatched bases were introduced to the probe and forward and reverse primers to reduce the generation of dimers between the probe and reverse primer. The reason for introducing mismatches is to ensure that the probe and reverse primer have fewer than three consecutive complementary bases, the complementary region of the probe and primer does not cover the THF site, the 3’ end of the reverse primer and probe does not have complementary pairing of more than three bases, and A-G and T-C are preferentially used interchangeably. The introduction of mismatches resulted in suitable probes and reverse primers. The primer sequences are listed in **Table 1**. As indicated by the experimental results presented in **Figures 1C, D**, the test line of the experimental group had an obvious red band, but not test line of the no template control group, indicating the absence of false-positive signals.

### Specificity Validation of RPA-LFS

**Figure 2:**
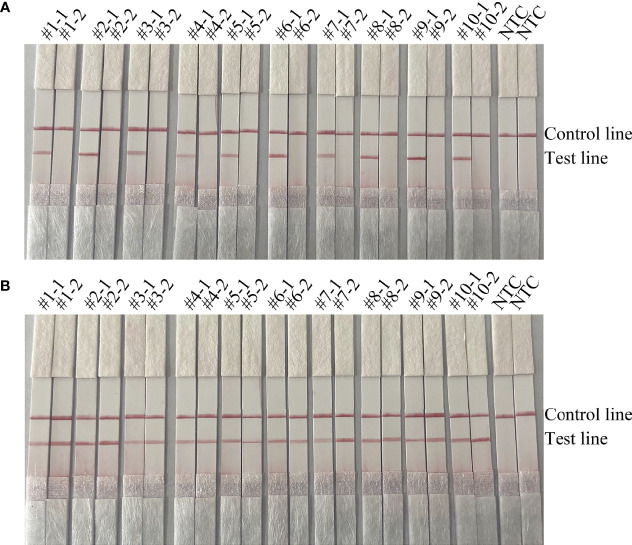
Validation of the specificity of the primer pairs *omp6*-F3/R1B/P and *bexA*-F3/R1B/P for capsulated and non-capsulated *H. influenzae*. -1 refers to the test results of *omp6*-F1/R1, −2 refers to the test results of *bexA*-F2/R2: **(A)** #1–#10 refer to the ten non-capsulated *H. influenzae* isolated from sputum; **(B)** #1–#10 refer to the ten capsulated *H. influenzae* isolates from sputum. NTC, no template control. The positions of the test and control lines are marked on the right of the strip image.

**Figure 3:**
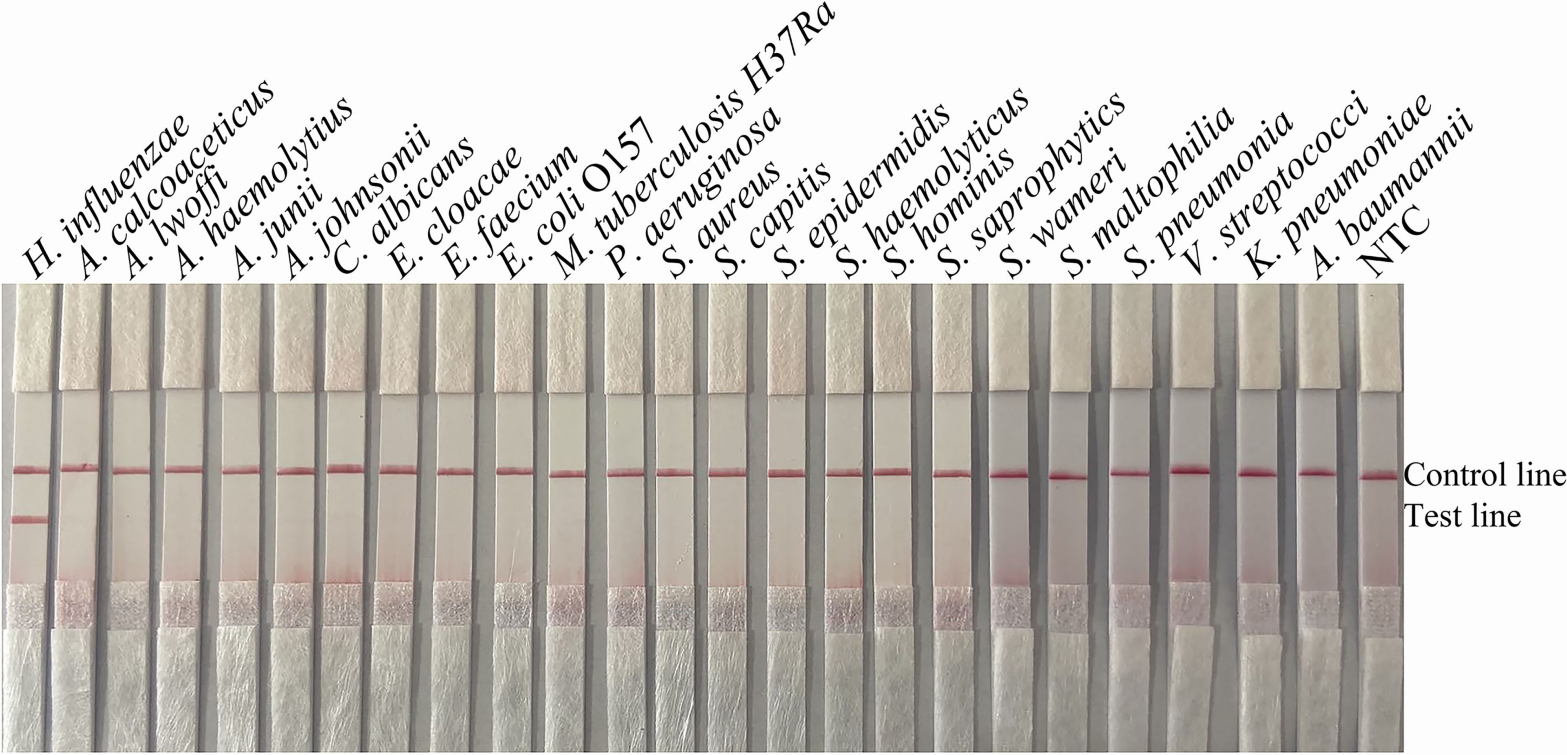
Specificity of *omp6*-F3/R1B/P. The template consisted of 1 µL of boiled bacterial culture at 10^6^ CFU/mL. Other pathogenic bacteria were tested. *H*. *influenzae* ATCC 49247 was used as a positive control. The species name is indicated at the top of each strip. NTC, no-template control. The positions of the test and control lines are marked on the right of the strip image. The reactions were performed for 30 min at 37°C. The images represent the results of three independent experiments.

To confirm that the primer pair *bexA*-F3/R1B/P only amplifies capsulated *H. influenzae* and that the primer pair *omp6*-F3/R1B/P only amplifies non-capsulated *H. influenzae*, specific primers corresponding to the two genes were used to amplify 10 non-capsulated and 10 capsulated *H. influenzae* isolates (verified by the traditional culture method). As shown in **Figures 2A, B**, *omp6*-F3/R1B/P detected all non-capsulated *H. influenzae*, while *bexA*-F3/R1B/P detected only capsulated *H. influenzae*, which confirmed the specificity of the primer pairs. In addition, to confirm that *omp6*-F3/R1B/P was specific to *H. influenzae*, 23 pathogens were selected for interspecies specificity verification (**Table 2**). The results showed that only *H. influenzae* produced red bands at both the test and control lines, while the other 23 pathogens produced red bands only at the control line (**Figure 3**).

**Table 2:** Bacterial strains used in the study.

Species	Source	RPA-LFS		Dual PCR		Culture-biochemical Methods
		*Omp6*	*bexA*_-_	*Omp6*	*bexA*	
Haemophilus influenzae (Non-Podoconiosis)	ATCC 49247	Positive	Negative	Positive	Negative	Positive
Haemophilus influenzae (Non-Podoconiosis)	Sputum isolated strain #1	Positive	Negative	Positive	Negative	Positive
Haemophilus influenzae (Non-Podoconiosis)	Sputum isolated strain #2	Positive	Negative	Positive	Negative	Positive
Haemophilus influenzae (Non-Podoconiosis)	Sputum isolated strain #3	Positive	Negative	Positive	Negative	Positive
Haemophilus influenzae (Non-Podoconiosis)	Sputum isolated strain #4	Positive	Negative	Positive	Negative	Positive
Haemophilus influenzae (Non-Podoconiosis)	Sputum isolated strain #5	Positive	Negative	Positive	Negative	Positive
Haemophilus influenzae (Non-Podoconiosis)	Sputum isolated strain #6	Positive	Negative	Positive	Negative	Positive
Haemophilus influenzae (Non-Podoconiosis)	Sputum isolated strain #7	Positive	Negative	Positive	Negative	Positive
Haemophilus influenzae (Non-Podoconiosis)	Sputum isolated strain #8	Positive	Negative	Positive	Negative	Positive
Haemophilus influenzae (Non-Podoconiosis)	Sputum isolated strain #9	Positive	Negative	Positive	Negative	Positive
Haemophilus influenzae (Non-Podoconiosis)	Sputum isolated strain #10	Positive	Negative	Positive	Negative	Positive
Haemophilus influenzae (Podoconiosis)	ATCC 9334	Positive	Positive	Positive	Positive	Positive
Haemophilus influenzae (Podoconiosis)	Sputum isolated strain #1	Positive	Positive	Positive	Positive	Positive
Haemophilus influenzae (Podoconiosis)	Sputum isolated strain #2	Positive	Positive	Positive	Positive	Positive
Haemophilus influenzae (Podoconiosis)	Sputum isolated strain #3	Positive	Positive	Positive	Positive	Positive
Haemophilus influenzae (Podoconiosis)	Sputum isolated strain #4	Positive	Positive	Positive	Positive	Positive
Haemophilus influenzae (Podoconiosis)	Sputum isolated strain #5	Positive	Positive	Positive	Positive	Positive
Haemophilus influenzae (Podoconiosis)	Sputum isolated strain #6	Positive	Positive	Positive	Positive	Positive
Haemophilus influenzae (Podoconiosis)	Sputum isolated strain #7	Positive	Positive	Positive	Positive	Positive
Haemophilus influenzae (Podoconiosis)	Sputum isolated strain #8	Positive	Positive	Positive	Positive	Positive
Haemophilus influenzae (Podoconiosis)	Sputum isolated strain #9	Positive	Positive	Positive	Positive	Positive
Haemophilus influenzae (Podoconiosis)	Sputum isolated strain #10	Positive	Positive	Positive	Positive	Positive
Acinetobacter calcoaceticus	Sputum isolated strain	Negative	Negative	Negative	Negative	Negative
Acinetobacter lwoffi	Sputum isolated strain	Negative	Negative	Negative	Negative	Negative
Acinetobacter haemolytius	Sputum isolated strain	Negative	Negative	Negative	Negative	Negative
Acinetobacter junii	Sputum isolated strain	Negative	Negative	Negative	Negative	Negative
Acinetobacter johnsonii	Sputum isolated strain	Negative	Negative	Negative	Negative	Negative
Candida albicans	ATCC 10231	Negative	Negative	Negative	Negative	Negative
Enterobacter cloacae	Sputum isolated strain	Negative	Negative	Negative	Negative	Negative
Enterococcus faecium	Sputum isolated strain	Negative	Negative	Negative	Negative	Negative
Escherichia coli O157	Sputum isolated strain	Negative	Negative	Negative	Negative	Negative
Mycobacterium tuberculosis H37Ra	Sputum isolated strain	Negative	Negative	Negative	Negative	Negative
Pseudomonas aeruginosa	Sputum isolated strain	Negative	Negative	Negative	Negative	Negative
Staphylococcus aureus	Sputum isolated strain	Negative	Negative	Negative	Negative	Negative
Staphylococcus capitis	Sputum isolated strain	Negative	Negative	Negative	Negative	Negative
Staphylococcus epidermidis	Sputum isolated strain	Negative	Negative	Negative	Negative	Negative
Staphylococcus haemolyticus	Sputum isolated strain	Negative	Negative	Negative	Negative	Negative
Staphylococcus hominis	Sputum isolated strain	Negative	Negative	Negative	Negative	Negative
Staphylococcus saprophytics	Sputum isolated strain	Negative	Negative	Negative	Negative	Negative
Staphylococcus wameri	Sputum isolated strain	Negative	Negative	Negative	Negative	Negative
Stenotrophomonas maltophilia	Sputum isolated strain	Negative	Negative	Negative	Negative	Negative
Streptococcus pneumonia	Sputum isolated strain	Negative	Negative	Negative	Negative	Negative
Viridans streptococci	Sputum isolated strain	Negative	Negative	Negative	Negative	Negative
Klebsiella pneumoniae	Sputum isolated strain	Negative	Negative	Negative	Negative	Negative
Acinetobacter baumannii	ATCC 19606	Negative	Negative	Negative	Negative	Negative

### Validation of the Detection Limit of RPA-LFS

**Figure 4:**
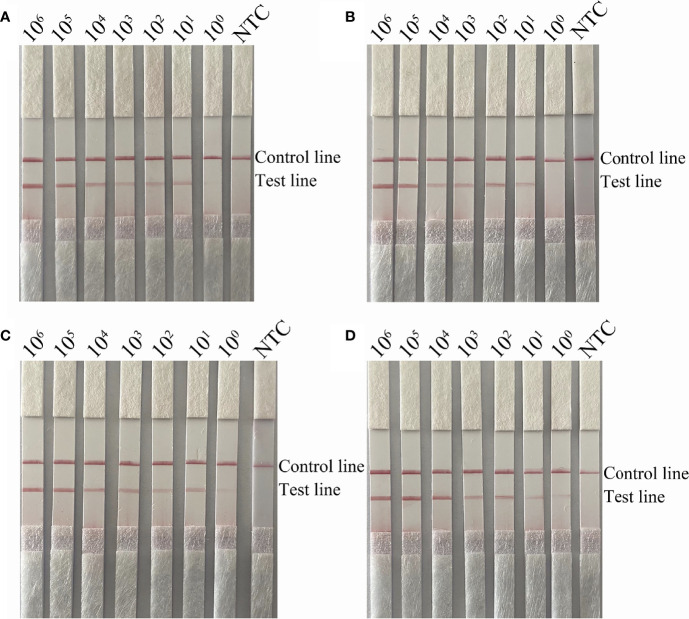
Detection limit of the RPA-LFS system. **(A, B)** LFS results of RPA amplifications with different amounts of non-capsulated *H. influenzae*. The amounts (in CFU) added to the RPA reactions are indicated at the top of each strip. In **(B)**, 10^5^ CFU/μL of *S. pneumoniae* were added to the reactions in addition to the non-capsulated *H. influenzae* culture. **(C, D)** LFS results of RPA amplifications with different amounts of capsulated *H. influenzae*. The amounts added to the RPA reactions are indicated at the top of each strip. In **(D)**, 10^5^ CFU/μL of the culture of *S. pneumoniae* was added to the reactions in addition to the capsulated *H. influenzae*. NTC, no template control. The reactions were performed for 30 min at 37°C. The positions of the control and test lines are indicated on the right of the images.

To verify the detection limit of the RPA-LFS assay, boiled cultures of the capsulated and non-capsulated *H. influenzae* were serially diluted. Briefly, 1 µL of diluted culture was added to each 50-mL reaction volume for a final concentration of ranging from 10^6^ to 10^0^ CFU/µL. As shown by the results presented in **Figures 4A, C**, the lowest detection limit for the primer pairs *omp6*-F3/R1B/P and *bexA*-F3/R1B/P was 10^0^ CFU/reaction, indicating that the RPA-LFS assay was more sensitive than the PCR assay. The addition of *S. pneumoniae* to different concentrations of *H. influenzae* had no effect on the sensitivity of the RPA-LFS assay, as the lowest detection limit remained at 10^0^ CFU (**Figures 4B, D**).

### Evaluation of the RPA-LFS Assay With Clinical Specimens

The practical application of the RPA-LFS assay was verified using 209 clinical *H*. *influenzae* samples. The results showed that 203 of the samples were correctly identified as *H. influenzae*, yielding a detection rate of 97.1%, which was consistent with the detection results of the dual PCR assay and traditional culture methods. In order to assess the specificity for capsulated *H. influenzae*, all samples were screened with the primer pair *bexA*-F3/R1B/P and the results were compared with those of the dual PCR assay. The results showed that 128 capsulated *H. influenzae* isolates were detected by both the RPA-LFS and dual PCR assays, yielding a detection rate of 63.2% (**Table 3**), demonstrating that the specificity of the RPA-LFS assay was equal to those of the dual PCR assay and traditional culture method.

**Table 3:** 209 strains using RPA-LFS and dual PCR.

Method	*Omp6*	N (%)	*bexA*	N (%)
RPA-LFS	203	97.1	128	63.2
Dual PCR	203	97.1	128	63.2
Coincidence rate(%)	N/A	100%	N/A	100%

N, number.

## Discussion

*H. influenzae* is a major cause of meningitis, sepsis, otitis media, and other diseases, and *H. influenzae* type b is the most pathogenic (Moxon and Vaughn, 1981; Saikia et al., 2011). The traditional culture method is the commonly used technique for detection of *H. influenzae*. However, *H. influenzae* is a fastidious bacterium, which requires special growth factors, an anaerobic environment, and a relatively long culture period. Since this process is rather complicated, it is not conducive to timely diagnosis and treatment (Devakanthan et al., 2021). PCR is currently the most widely used technology for detection of nucleic acids. Although PCR has been successfully applied for the detection of *H. influenzae*, this technology requires expensive precision instruments and professional technicians, which are typically available only in hospitals and key laboratories (Marty et al., 2004).

Current nucleic acid detection technologies allow for amplification at a constant temperature. Cao et al. (2021) successfully established a thermostatic amplification technology for the detection of *H. influenzae* based on the *omp6* gene using multiple cross displacement amplification and a nanoparticle-based lateral flow biosensor. However, the primer design is complex and prone to false-positive results (Dong et al., 2020). Recombinase polymerase amplification technology allows for amplification at a constant temperature of 30–45°C, with relatively higher sensitivity and specificity, simple primer design, and no need for expensive equipment. In addition, RPA has a certain tolerance, and the detection can be achieved only by using inactivated bacterial liquid, without the need for the use of expensive and complicated genome extraction kits. In this study, false-positive results due to the formation of primer dimers were also avoided by introducing mismatches between the probes and primers, which had no effect on the amplification efficiency of the primers.

In order to accurately determine the type of *H. influenzae* and facilitate subsequent antibiotic treatment, specific forward and reverse primers and probes were designed based on the *omp6* and *bexA* genes, respectively (Fan et al., 2018). The specificity of the primers for the *omp6* gene was verified and no cross-amplification of other strains was observed. In order to verify the sensitivity of the two primer pairs, standard capsulated and non-capsulated *H. influenzae* were detected, and an equal amount of *S*. *pneumoniae* (10^5^ CFU) was added a solution of inactivated *H*. *influenzae* at different concentrations. The lowest limit of detection of the proposed RPA-LFS assay was 1 CFU of *H. influenzae* and the addition of other pathogens did not interfere with the detection sensitivity. The detection of clinical samples confirmed that the specificity of the assay was consistent with that of the dual PCR assay. Of the 209 clinical samples, 203 were correctly identified as *H. influenzae* and 128 as capsulated *H. influenzae*, yielding detection rates of 97.1% and 63.2%, respectively. The applicability of the RPA-LFS assay is equal to that of the dual PCR assay. In this study, an RPA-LFS assay was successfully established based on the *omp6* and *bexA* genes for the detection of capsulated and non-capsulated *H. influenzae*. This assay allows for rapid detection of *H. influenzae* to ensure timely diagnosis and treatment of patients, and hopefully meet the need for field testing in remote resource-poor areas.

## Conclusion

The RPA–LFS assay was simple, rapid, and highly specific. An improved RPA assay for visual detection of capsulated and non-capsulated *H. influenza* with LFS was developed. False-positive signals on the test strip caused by primer-dimers were successfully ruled out by introducing mismatched bases into the probes and primers. The method to distinguish capsulated and non-capsulated *H. influenzae* provides a solution for the rapid diagnosis of infectious diseases and the corresponding antibiotic treatment.

## Data Availability Statement

The original contributions presented in the study are included in the article/supplementary material. Further inquiries can be directed to the corresponding authors.

## Author Contributions

YW, LW, and FFM designed the experiments and wrote the manuscript. ABL, JJG, and XHZ collected the clinical samples. YW and MF performed the main experiments. LW analyzed the data. All authors contributed to the article and approved the submitted version.

## Funding

This study was supported by grants from the Jiangsu University Clinical Medicine Science and Technology Development Fund Project (grant number JLY2021088), the Lianyungang City Health Science and Technology Project (grant number 202122), the Lianyungang Science and Technology Bureau, Municipal Science and Technology Plan (Social Development) Project (grant number SF2140).

## Conflict of Interest

The authors declare that the research was conducted in the absence of any commercial or financial relationships that could be construed as a potential conflict of interest.

## Publisher’s Note

All claims expressed in this article are solely those of the authors and do not necessarily represent those of their affiliated organizations, or those of the publisher, the editors and the reviewers. Any product that may be evaluated in this article, or claim that may be made by its manufacturer, is not guaranteed or endorsed by the publisher.

## References

[B1] Abdeldaim G. M. Strålin K. Korsgaard J. Blomberg J. Welinder-Olsson C. Herrmann B. (2010). Multiplex Quantitative PCR for Detection of Lower Respiratory Tract Infection and Meningitis Caused by Streptococcus Pneumoniae, Haemophilus Influenzae and Neisseria Meningitidis. BMC Microbiol. 10, 310. doi: 10.1186/1471-2180-10-310 21129171 PMC3016321

[B2] Adegbola R. A. DeAntonio R. Hill P. C. Roca A. Usuf E. Hoet B.. (2014). Carriage of Streptococcus Pneumoniae and Other Respiratory Bacterial Pathogens in Low and Lower-Middle Income Countries: A Systematic Review and Meta-Analysis. PloS One 9 (8), e103293. doi: 10.1371/journal.pone.0103293 25084351 PMC4118866

[B3] Ali M. Chang B. A. Johnson K. W. Morris S. K. (2018). Incidence and Aetiology of Bacterial Meningitis Among Children Aged 1-59 Months in South Asia: Systematic Review and Meta-Analysis. Vaccine 36 (39), 5846–5857. doi: 10.1016/j.vaccine.2018.07.037 30145101

[B4] Biondi E. A. Lee B. Ralston S. L. Winikor J. M. Lynn J. F. Dixon A.. (2019). Prevalence of Bacteremia and Bacterial Meningitis in Febrile Neonates and Infants in the Second Month of Life: A Systematic Review and Meta-Analysis. JAMA Netw. Open 2 (3), e190874. doi: 10.1001/jamanetworkopen.2019.0874 30901044 PMC6583289

[B5] Boukharouba A. González A. García-Ferrús M. Ferrús M. A. Botella S. (2022). Simultaneous Detection of Four Main Foodborne Pathogens in Ready-To-Eat Food by Using a Simple and Rapid Multiplex PCR (mPCR) Assay. Int. J. Environ. Res. Public Health 19, (3). doi: 10.3390/ijerph19031031 PMC8834630 35162055

[B6] Cao Q. Liang S. Wang L. Cao J. Liu M. Li S.. (2021). A Rapid Detection of Haemophilus Influenzae Using Multiple Cross Displacement Amplification Linked With Nanoparticle-Based Lateral Flow Biosensor. Front. Cell. Infect. Microbiol. 11, 721547. doi: 10.3389/fcimb.2021.721547 34631602 PMC8493954

[B7] Daher R. K. Stewart G. Boissinot M. Bergeron M. G. (2016). Recombinase Polymerase Amplification for Diagnostic Applications. Clin. Chem. 62 (7), 947–958. doi: 10.1373/clinchem.2015.245829 27160000 PMC7108464

[B8] Devakanthan B. Liyanapathirana V. Dissanayake N. Harasgama P. Punchihewa J. (2021). Identification of Bacterial Aetiology in Acute Meningitis. Ceylon Med. J. 66 (2), 65–72. doi: 10.4038/cmj.v66i2.9465 34989220

[B9] Dong Y. Zhao P. Chen L. Wu H. Si X. Shen X.. (2020). Fast, Simple and Highly Specific Molecular Detection of Vibrio Alginolyticus Pathogenic Strains Using a Visualized Isothermal Amplification Method. BMC Veterinary Res. 16 (1), 76. doi: 10.1186/s12917-020-02297-4 PMC7057676 32131821

[B10] Fan X. Liu X. Ji L. Cai D. Jiang J. Zhu J.. (2018). Epidemiological Analysis and Rapid Detection by One-Step Multiplex PCR Assay of Haemophilus Influenzae in Children With Respiratory Tract Infections in Zhejiang Province, China. BMC Infect. Dis. 18 (1), 414. doi: 10.1186/s12879-018-3295-2 30134854 PMC6103868

[B11] Farajzadeh Sheikh A. Rahimi R. Meghdadi H. Alami A. Saki M. (2021). Multiplex Polymerase Chain Reaction Detection of Streptococcus Pneumoniae and Haemophilus Influenzae and Their Antibiotic Resistance in Patients With Community-Acquired Pneumonia From Southwest Iran. BMC Microbiol. 21 (1), 343. doi: 10.1186/s12866-021-02408-7 34906085 PMC8670030

[B12] Fuji N. Pichichero M. Kaur R. (2021). Haemophilus Influenzae Prevalence, Proportion of Capsulated Strains and Antibiotic Susceptibility During Colonization and Acute Otitis Media in Childre-2020. Pediatr. Infect. Dis. J. 40 (9), 792–796. doi: 10.1097/inf.0000000000003171 34321442

[B13] Gessner B. D. (2002). Worldwide Variation in the Incidence of Haemophilus Influenzae Type B Meningitis and Its Association With Ampicillin Resistance. Eur. J. Clin. Microbiol. Infect. Dis.: Off. Publ. Eur. Soc. Clin. Microbiol. 21 (2), 79–87. doi: 10.1007/s10096-001-0667-z 11939404

[B14] Giufrè M. Dorrucci M. Lo Presti A. Farchi F. Cardines R. Camilli R.. (2022). Nasopharyngeal Carriage of Haemophilus Influenzae Among Adults With Co-Morbidities. Vaccine 40 (5), 826–832. doi: 10.1016/j.vaccine.2021.12.030 34952754

[B15] Guellil M. Keller M. Dittmar J. M. Inskip S. A. Cessford C. Solnik A.. (2022). An Invasive Haemophilus Influenzae Serotype B Infection in an Anglo-Saxon Plague Victim. Genome Biol. 23 (1), 22. doi: 10.1186/s13059-021-02580-z 35109894 PMC8812261

[B16] Langereis J. D. de Jonge M. I. (2015). Invasive Disease Caused by Nontypeable Haemophilus Influenzae. Emerg. Infect. Dis. 21 (10), 1711–1718. doi: 10.3201/eid2110.150004 26407156 PMC4593434

[B17] Liu X. Yan Q. Huang J. Chen J. Guo Z. Liu Z.. (2019). Influence of Design Probe and Sequence Mismatches on the Efficiency of Fluorescent RPA. World J. Microbiol. Biotechnol. 35 (6), 95. doi: 10.1007/s11274-019-2620-2 31187258

[B18] Marty A. Greiner O. Day P. J. Gunziger S. Mühlemann K. Nadal D. (2004). Detection of Haemophilus Influenzae Type B by Real-Time PCR. J. Clin. Microbiol. 42 (8), 3813–3815. doi: 10.1128/jcm.42.8.3813-3815.2004 15297536 PMC497579

[B19] Moxon E. R. Kroll J. S. (1988). Type B Capsular Polysaccharide as a Virulence Factor of Haemophilus Influenzae. Vaccine 6 (2), 113–115. doi: 10.1016/s0264-410x(88)80011-2 3291448

[B20] Moxon E. R. Vaughn K. A. (1981). The Type B Capsular Polysaccharide as a Virulence Determinant of Haemophilus Influenzae: Studies Using Clinical Isolates and Laboratory Transformants. J. Infect. Dis. 143 (4), 517–524. doi: 10.1093/infdis/143.4.517 6972419

[B21] Nolen L. D. Topaz N. Miernyk K. Bressler S. Massay S. C. Geist M.. (2022). Evaluating a Cluster and the Overall Trend of Invasive Haemophilus Influenzae Serotype B in Alaska 2005-2019. Pediatr. Infect. Dis. J. 41 (4), 120–125. doi: 10.1097/inf.0000000000003470 35067639

[B22] Piepenburg O. Williams C. H. Stemple D. L. Armes N. A. (2006). DNA Detection Using Recombination Proteins. PloS Biol. 4 (7), e204. doi: 10.1371/journal.pbio.0040204 16756388 PMC1475771

[B23] Saikia K. K. Bewal R. Bansal D. Kapil A. Sood S. Arora N. K.. (2011). Multi Locus Sequence Type Comparison of Invasive and Commensal Haemophilus Influenzae Isolates From Delhi. Indian J. Med. Microbiol. 29 (2), 158–160. doi: 10.4103/0255-0857.81800 21654111

[B24] Serigstad S. Markussen D. Grewal H. M. S. Ebbesen M. Kommedal Ø Heggelund L.. (2022). Rapid Syndromic PCR Testing in Patients With Respiratory Tract Infections Reduces Time to Results and Improves Microbial Yield. Sci. Rep. 12 (1), 326. doi: 10.1038/s41598-021-03741-7 35013351 PMC8748978

[B25] Silva M. D. Lima A. Marçal N. Dias L. Gama M. Sillankorva S. (2021). Identification of the Bacterial Pathogens in Children With Otitis Media: A Study in the Northwestern Portuguese District of Braga. Microorganisms 10 (1), 54. doi: 10.3390/microorganisms10010054 35056502 PMC8779683

[B26] Takahashi Y. Osawa R. Kubota Y. Fujii M. Matsuda N. Watanabe N.. (2021). Early Diagnosis of Cryptococcus Neoformans Var. Grubii Meningitis Using Multiplex PCR Assay in an Immunocompetent Patient. J. Infect. Chemother.: Off. J. Japan Soc. Chemother. 27 (12), 1765–1768. doi: 10.1016/j.jiac.2021.08.006 34393039

[B27] Tian G. Z. Shao Z. J. Zhang L. Li X. J. Zhu B. Q. Yang Y. J.. (2008). Detection of Haemophilus Influenzae by Multiplex Polymerase Chain Reaction Method. Zhonghua Liu Xing Bing Xue Za Zhi Zhonghua Liuxingbingxue Zazhi 29 (8), 806–809. 19103119

[B28] Wang F. Ge D. Wang L. Li N. Chen H. Zhang Z.. (2021a). Rapid and Sensitive Recombinase Polymerase Amplification Combined With Lateral Flow Strips for Detecting Candida Albicans. Analytical Biochem. 633, 114428. doi: 10.1016/j.ab.2021.114428 34678249

[B29] Wang L. Wang Y. Wang F. Zhao M. Gao X. Chen H.. (2021b). Development and Application of Rapid Clinical Visualization Molecular Diagnostic Technology for Cryptococcus Neoformans/C. Gattii Based on Recombinase Polymerase Amplification Combined With a Lateral Flow Strip. Front. Cell. Infect. Microbiol. 11, 803798. doi: 10.3389/fcimb.2021.803798 35096653 PMC8790172

[B30] Wang L. Zhao P. Si X. Li J. Dai X. Zhang K.. (2019). Rapid and Specific Detection of Listeria Monocytogenes With an Isothermal Amplification and Lateral Flow Strip Combined Method That Eliminates False-Positive Signals From Primer-Dimers. Front. Microbiol. 10, 2959. doi: 10.3389/fmicb.2019.02959 32117075 PMC7025549

[B31] Wen S. Feng D. Chen D. Yang L. Xu Z. (2020). Molecular Epidemiology and Evolution of Haemophilus Influenzae. Infect. Genet. Evol.: J. Mol. Epidemiol. Evol. Genet. Infect. Dis. 80, 104205. doi: 10.1016/j.meegid.2020.104205 31981610

[B32] Wu H. Zhao P. Yang X. Li J. Zhang J. Zhang X.. (2020). A Recombinase Polymerase Amplification and Lateral Flow Strip Combined Method That Detects Salmonella Enterica Serotype Typhimurium With No Worry of Primer-Dependent Artifacts. Front. Microbiol. 11, 1015. doi: 10.3389/fmicb.2020.01015 32655504 PMC7324538

[B33] Yang X. Dong Y. Ma C. Qiao Y. Jiang G. Chen S.. (2021b). Establishment of a Visualized Isothermal Nucleic Acid Amplification Method for on-Site Diagnosis of Acute Hepatopancreatic Necrosis Disease in Shrimp Farm. J. Fish Dis. 44 (9), 1293–1303. doi: 10.1111/jfd.13388 34041767

[B34] Yang H. Wang Y. Yang Q. Fan H. Wang L. Zhang T.. (2021a). A Rapid and Sensitive Detection Method for Pseudomonas Aeruginosa Using Visualized Recombinase Polymerase Amplification and Lateral Flow Strip Technology. Front. Cell. Infect. Microbiol. 11, 698929. doi: 10.3389/fcimb.2021.698929 34595129 PMC8478171

[B35] Yang X. Zhao P. Dong Y. Shen X. Shen H. Li J.. (2020). An Improved Recombinase Polymerase Amplification Assay for Visual Detection of Vibrio Parahaemolyticus With Lateral Flow Strips. J. Food Sci. 85 (6), 1834–1844. doi: 10.1111/1750-3841.15105 32449955

[B36] Zhao Y. Wang R. Zhu W. Jin H. Fan X. (2017). Establishment and Clinical Application of Dual PCR Detection Methods for Capsulated and Non-Capsulated Haemophilus Influenzae China. J. Health Lab. Tec. 27 (21), 3102–3105.

